# 4-Carb­oxy­pyridinium bromide

**DOI:** 10.1107/S1600536810020209

**Published:** 2010-06-05

**Authors:** Yingchun Wang

**Affiliations:** a Ordered Matter Science Research Center, Southeast University, Nanjing 210096, People’s Republic of China

## Abstract

In the title compound, C_6_H_6_NO_2_^+^·Br^−^, the hy­droxy and carbonyl groups make torsion angles of 164.8 (4) and −17.6 (6)°, respectively, with the pyridinium ring. Inter­molecular N—H⋯Br, O—H⋯Br and C—H⋯O hydrogen bonds contribute to the stability of the structure and link the mol­ecules into chains along the *b* axis.

## Related literature

For the phase transition of pyridinium tetra­chloro­iodate(III) studied by X-ray analysis and for dielectric and heat capacity measurements, see: Asaji *et al.* (2007 ▶). For the ferroelecric properties of pyridinum perrhenate, see: Wasicki *et al.* (1997 ▶). For the structure of 3-carb­oxy­pyridinium chloride, see: Slouf (2001 ▶).
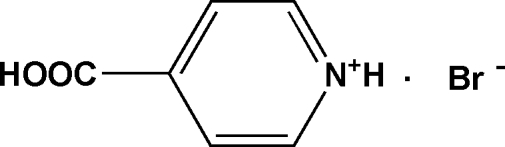


## Experimental

### 

#### Crystal data

C_6_H_6_NO_2_^+^·Br^−^
*M* *_r_* = 204.03
Monoclinic, 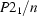

*a* = 7.3179 (15) Å
*b* = 7.3433 (15) Å
*c* = 13.532 (3) Å
β = 94.37 (3)°
*V* = 725.1 (3) Å^3^
*Z* = 4
Mo *K*α radiation
μ = 5.60 mm^−1^
*T* = 293 K
0.20 × 0.20 × 0.20 mm

#### Data collection

Rigaku SCXmini diffractometer
Absorption correction: multi-scan (*CrystalClear*; Rigaku, 2005 ▶) *T*_min_ = 0.326, *T*_max_ = 0.339
7062 measured reflections
1670 independent reflections
1167 reflections with *I* > 2σ(*I*)
*R*_int_ = 0.073

#### Refinement

*R*[*F* ^2^ > 2σ(*F* ^2^)] = 0.044
*wR*(*F* ^2^) = 0.097
*S* = 1.06
1670 reflections
91 parameters
H-atom parameters constrained
Δρ_max_ = 0.45 e Å^−3^
Δρ_min_ = −0.33 e Å^−3^

### 

Data collection: *CrystalClear* (Rigaku, 2005 ▶); cell refinement: *CrystalClear*; data reduction: *CrystalClear*; program(s) used to solve structure: *SHELXS97* (Sheldrick, 2008 ▶); program(s) used to refine structure: *SHELXL97* (Sheldrick, 2008 ▶); molecular graphics: *SHELXTL* (Sheldrick, 2008 ▶); software used to prepare material for publication: *PRPKAPPA* (Ferguson, 1999 ▶) and *PLATON* (Spek, 2009 ▶).

## Supplementary Material

Crystal structure: contains datablocks I, global. DOI: 10.1107/S1600536810020209/si2263sup1.cif

Structure factors: contains datablocks I. DOI: 10.1107/S1600536810020209/si2263Isup2.hkl

Additional supplementary materials:  crystallographic information; 3D view; checkCIF report

## Figures and Tables

**Table 1:** Hydrogen-bond geometry (Å, °)

*D*—H⋯*A*	*D*—H	H⋯*A*	*D*⋯*A*	*D*—H⋯*A*
O2—H2*A*⋯Br1^i^	0.82	2.32	3.127 (3)	170
N1—H1*A*⋯Br1^ii^	0.86	2.45	3.253 (3)	155
C4—H4*A*⋯O1^iii^	0.93	2.39	3.044 (5)	127

Symmetry codes: (i) 
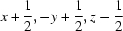
; (ii) 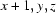; (iii) 
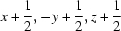
.

**Fig. 1.**
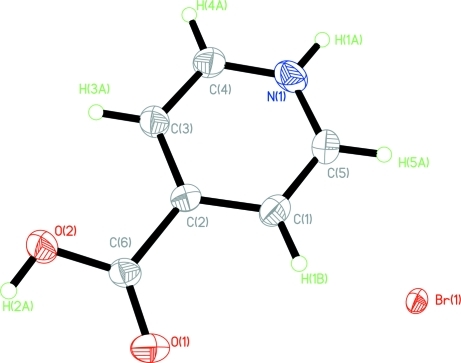
The molecular structure of the title compound, with the atomic numbering scheme. Displacement ellipsoids are drawn at the 30% probability level.

**Fig. 2.**
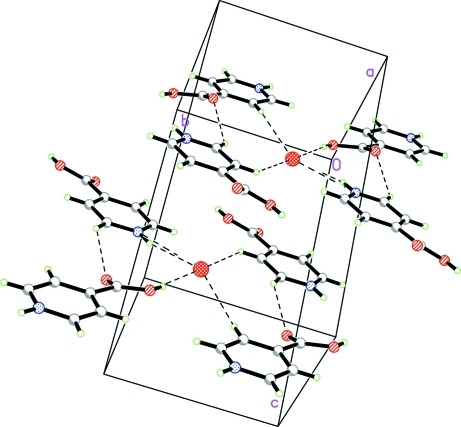
A view of a packing section of the title compound. Dashed lines indicate hydrogen bonds.

## supplementary crystallographic information

### Comment

Some materials have predominant dielectric-ferroelectric performance and they have much applications in many fields. The study of dielectric-ferroelectric materials has received much attention in recent years. PyHX(*X*=ICl_4_,ClO_4_,IO_4_,ReO_4_*etc*) (Asaji *et al.*(2007); Wasicki *et al.*(1997)) are representative. As one part of our continuing studies on finding for dielectric-ferroelectric materials, we synthesized the title compound C_6_H_6_NO_2_^+^Br^-^ unexpected comparing to PyHX, but it has no phase-transition in dielectric-ferroelectric measurement during 93 K to 470 K (m.p. 483 K).

The asymmetric unit of the title compound contains one 4-Carboxypyridinium basic ion and one bromide negative ion (Fig 1). In contrast to the planar 3-carboxypyridinium chloride (Slouf, 2001), the carboxyl group in the title molecule is slightly rotated with torsion angles of 164.8 (4)° and -17.6 (6)°. In the planar 3-carboxypyridinium chloride structure, N—H···O hydrogen bonds form chains along the *c* axis, whereas in the title structure, 4-Carboxypyridinium basic ions and bromide ions are linked into chains along *b* through hydrogen bonds (Table 1, Fig 2). Crystallographic details of the title structure were examined with PLATON (Spek, 2009).

### Experimental

Methyl benzoate 0.685 g (5 mmol) in ethanol (30 ml), and 1.01 g hydrobromic acid (40%, 5 mmol) was added. The mixed solution was filtrated and the crystals suitable for structure determination were grown by slow evaporation of the solution at room temperature for five days.

### Refinement

Positional parameters of all the H atoms were calculated geometrically and were allowed to ride on the C atoms to which they are bonded,with C—H = 0.93 Å, N—H = 0.75–0.86 Å; with *U*_iso_(H) = 1.2*U*_eq_(C), with *U*_iso_(H) = 1.2–1.5*U*_eq_(N).

### Crystal data

**Table:**

C_6_H_6_NO_2_^+^·Br^−^	*F*(000) = 400
*M**_r_* = 204.03	*D*_x_ = 1.869 Mg m^−^^3^
Monoclinic, *P*2_1_/*n*	Mo *K*α radiation, λ = 0.71073 Å
Hall symbol: -P 2yn	Cell parameters from 0 reflections
*a* = 7.3179 (15) Å	θ = 3.0–27.5°
*b* = 7.3433 (15) Å	µ = 5.60 mm^−^^1^
*c* = 13.532 (3) Å	*T* = 293 K
β = 94.37 (3)°	Prism, colorless
*V* = 725.1 (3) Å^3^	0.20 × 0.20 × 0.20 mm
*Z* = 4	

### Data collection

**Table:**

Rigaku SCXmini diffractometer	1670 independent reflections
Radiation source: fine-focus sealed tube	1167 reflections with *I* > 2σ(*I*)
graphite	*R*_int_ = 0.073
Detector resolution: 13.6612 pixels mm^-1^	θ_max_ = 27.5°, θ_min_ = 3.0°
CCD_Profile_fitting scans	*h* = −9→9
Absorption correction: multi-scan (*CrystalClear*; Rigaku, 2005)	*k* = −9→9
*T*_min_ = 0.326, *T*_max_ = 0.339	*l* = −17→17
7062 measured reflections	

### Refinement

**Table:**

Refinement on *F*^2^	Primary atom site location: structure-invariant direct methods
Least-squares matrix: full	Secondary atom site location: difference Fourier map
*R*[*F*^2^ > 2σ(*F*^2^)] = 0.044	Hydrogen site location: inferred from neighbouring sites
*wR*(*F*^2^) = 0.097	H-atom parameters constrained
*S* = 1.06	*w* = 1/[σ^2^(*F*_o_^2^) + (0.0363*P*)^2^] where *P* = (*F*_o_^2^ + 2*F*_c_^2^)/3
1670 reflections	(Δ/σ)_max_ < 0.001
91 parameters	Δρ_max_ = 0.45 e Å^−^^3^
0 restraints	Δρ_min_ = −0.33 e Å^−^^3^

### Special details

**Table:**

Geometry. All e.s.d.'s (except the e.s.d. in the dihedral angle between two l.s. planes) are estimated using the full covariance matrix. The cell e.s.d.'s are taken into account individually in the estimation of e.s.d.'s in distances, angles and torsion angles; correlations between e.s.d.'s in cell parameters are only used when they are defined by crystal symmetry. An approximate (isotropic) treatment of cell e.s.d.'s is used for estimating e.s.d.'s involving l.s. planes.
Refinement. Refinement of *F*^2^ against ALL reflections. The weighted *R*-factor *wR* and goodness of fit *S* are based on *F*^2^, conventional *R*-factors *R* are based on *F*, with *F* set to zero for negative *F*^2^. The threshold expression of *F*^2^ > σ(*F*^2^) is used only for calculating *R*-factors(gt) *etc*. and is not relevant to the choice of reflections for refinement. *R*-factors based on *F*^2^ are statistically about twice as large as those based on *F*, and *R*- factors based on ALL data will be even larger.

### Fractional atomic coordinates and isotropic or equivalent isotropic displacement parameters (Å^2^)

**Table:**

	*x*	*y*	*z*	*U*_iso_*/*U*_eq_	
Br1	0.05149 (7)	0.81361 (5)	0.25911 (3)	0.0471 (2)	
O2	0.7136 (4)	−0.0655 (4)	−0.06801 (19)	0.0495 (8)	
H2A	0.6581	−0.1279	−0.1107	0.074*	
C2	0.7434 (6)	0.2225 (5)	0.0056 (3)	0.0351 (9)	
O1	0.5215 (4)	0.1518 (4)	−0.1253 (2)	0.0534 (9)	
N1	0.8996 (5)	0.4472 (5)	0.1454 (3)	0.0501 (10)	
H1A	0.9504	0.5186	0.1897	0.060*	
C1	0.7207 (6)	0.4094 (5)	−0.0030 (3)	0.0470 (11)	
H1B	0.6524	0.4587	−0.0573	0.056*	
C5	0.7998 (6)	0.5208 (6)	0.0693 (3)	0.0495 (12)	
H5A	0.7840	0.6463	0.0653	0.059*	
C3	0.8476 (6)	0.1523 (5)	0.0853 (3)	0.0450 (12)	
H3A	0.8657	0.0273	0.0912	0.054*	
C6	0.6474 (6)	0.0997 (5)	−0.0713 (3)	0.0375 (10)	
C4	0.9248 (7)	0.2683 (6)	0.1562 (3)	0.0498 (12)	
H4A	0.9938	0.2226	0.2113	0.060*	

### Atomic displacement parameters (Å^2^)

**Table:**

	*U*^11^	*U*^22^	*U*^33^	*U*^12^	*U*^13^	*U*^23^
Br1	0.0633 (4)	0.0335 (2)	0.0427 (3)	0.0000 (2)	−0.0077 (2)	0.0051 (2)
O2	0.067 (2)	0.0397 (17)	0.0395 (17)	0.0007 (15)	−0.0115 (16)	−0.0047 (14)
C2	0.034 (2)	0.039 (2)	0.033 (2)	0.0015 (17)	0.0009 (18)	0.0023 (18)
O1	0.049 (2)	0.061 (2)	0.0477 (19)	0.0024 (15)	−0.0130 (17)	−0.0010 (15)
N1	0.054 (3)	0.050 (2)	0.046 (2)	−0.0040 (19)	−0.0036 (19)	−0.0168 (19)
C1	0.053 (3)	0.041 (2)	0.046 (3)	0.008 (2)	−0.006 (2)	0.004 (2)
C5	0.053 (3)	0.035 (2)	0.060 (3)	0.000 (2)	0.000 (3)	0.003 (2)
C3	0.057 (3)	0.038 (2)	0.039 (3)	−0.001 (2)	−0.006 (2)	0.0000 (19)
C6	0.044 (3)	0.041 (2)	0.027 (2)	−0.001 (2)	−0.001 (2)	0.0008 (18)
C4	0.060 (3)	0.048 (3)	0.040 (3)	0.005 (2)	−0.012 (2)	−0.002 (2)

### Geometric parameters (Å, °)

**Table:**

O2—C6	1.306 (5)	N1—H1A	0.8600
O2—H2A	0.8200	C1—C5	1.369 (6)
C2—C3	1.373 (5)	C1—H1B	0.9300
C2—C1	1.386 (5)	C5—H5A	0.9300
C2—C6	1.508 (5)	C3—C4	1.372 (6)
O1—C6	1.195 (5)	C3—H3A	0.9300
N1—C5	1.330 (5)	C4—H4A	0.9300
N1—C4	1.333 (5)		
			
C6—O2—H2A	109.5	N1—C5—H5A	120.4
C3—C2—C1	119.6 (4)	C1—C5—H5A	120.4
C3—C2—C6	121.2 (3)	C4—C3—C2	119.4 (4)
C1—C2—C6	119.2 (4)	C4—C3—H3A	120.3
C5—N1—C4	123.3 (4)	C2—C3—H3A	120.3
C5—N1—H1A	118.4	O1—C6—O2	125.8 (4)
C4—N1—H1A	118.4	O1—C6—C2	121.8 (4)
C5—C1—C2	119.3 (4)	O2—C6—C2	112.3 (4)
C5—C1—H1B	120.3	N1—C4—C3	119.2 (4)
C2—C1—H1B	120.3	N1—C4—H4A	120.4
N1—C5—C1	119.2 (4)	C3—C4—H4A	120.4
			
C3—C2—C1—C5	−1.2 (6)	C3—C2—C6—O1	160.3 (4)
C6—C2—C1—C5	176.6 (4)	C1—C2—C6—O1	−17.6 (6)
C4—N1—C5—C1	−1.2 (7)	C3—C2—C6—O2	−17.4 (6)
C2—C1—C5—N1	1.2 (7)	C1—C2—C6—O2	164.8 (4)
C1—C2—C3—C4	1.2 (7)	C5—N1—C4—C3	1.2 (7)
C6—C2—C3—C4	−176.6 (4)	C2—C3—C4—N1	−1.2 (7)

### Hydrogen-bond geometry (Å, °)

**Table:**

*D*—H···*A*	*D*—H	H···*A*	*D*···*A*	*D*—H···*A*
O2—H2A···Br1^i^	0.82	2.32	3.127 (3)	170
N1—H1A···Br1^ii^	0.86	2.45	3.253 (3)	155
C4—H4A···O1^iii^	0.93	2.39	3.044 (5)	127

Symmetry codes: (i) *x*+1/2, −*y*+1/2, *z*−1/2; (ii) *x*+1, *y*, *z*; (iii) *x*+1/2, −*y*+1/2, *z*+1/2.
